# PEComa of the Uterus: A Rare Mesenchymal Tumor Displaying a ≪Snowstorm≫ Pattern at Magnetic Resonance Imaging

**DOI:** 10.5334/jbr-btr.926

**Published:** 2016-01-29

**Authors:** N. Verbeeck, A. Toukouki, R. Weis, D. Van Wymersch

**Affiliations:** 1 Department of Radiology, Centre Hospitalier de Luxembourg, rue Barblé, 4, L-1210 Luxembourg, Grand Duchy of Luxembourg; 2 Department of Obstetrics and Gynecology, Centre Hospitalier du Nord, avenue Salentiny, 120, L-9080 Ettelbruck, Grand Duchy of Luxembourg; 3 Department of Obsterics and Gynecology, Centre Hospitalier de Luxembourg, rue Barblé, 4, L-1210 Luxembourg, Grand Duchy of Luxembourg

**Keywords:** tumor, pecoma, ultrasound, Doppler, magnetic resonance

Dear Editor in Chief,

PEComa is an uncommon mesenchymal tumor with about 100 cases described in the literature up to 2014 [1,2,3]. If the first mention of this type of lesion dates back to 1943, when Apiz described an « abnormal myoblast », the normal counterpart cell of the lesions has remained unknown up to now [2,3,4]. The tumors are composed of epithelioid cells or, more seldom, of spindle cells, sometimes mixed in variable proportions and showing numerous analogies with those of the vascular walls, their probable site of origin [2,3]. As soon as 1992, Bonetti et al. suggested the term perivascular epithelioid cell tumor or PEComa to identify these lesions, whose aggressiveness proves variable [3].

The World Health Organization defines PEComa as a mesenchymal tumor composed of perivascular epithelioid cells with specific histologic and immunohistochemical features [3]. This definition applies to a family of neoplasms of variable anatomical topography including angiomyolipomas, clear cell sugar tumors, lymphangioleiomyomatosis and melanocytic tumors of the falciform ligament [1,2,3].

The histologic and immunohistochemical features of the tumors suggest that they emerge from the vascular parietal structures even if the corresponding normal cell is still not clearly identified (3). Indeed, PEComas consist of variable assemblies of epithelioid cells and, to a lesser extent, of spindle cells expressing a myomelanocytic phenotype, immunoreactive for melanocytic and smooth muscle markers like HMB-45 and smooth muscle actin in particular [1,2,3].

PEComas occur at any age, with a peak in the 4^th^ decade of life, affecting principally the female gender [1,2,3,4,5,6,7]. Case descriptions of this type have been increasing since 2008 and the majority (approximately 65%) involving the retroperitoneum, the kidneys and the genitourinary tract [1,2,3]. Other locations exist, such as liver, lung, pancreas, small bowel or colon but they are even much rarer [2,4,5,8]. Uterine PEComas are mainly located at the level of the corpus of the organ, the cervix being less often affected. 10% of the cases are associated with Bourneville tuberous sclerosis [1,2,3]. This association could be due to genetic mutations causing the inactivation of TSC1 or TSC2 genes [3].

The tumor aggressiveness of PEComas remains ill-defined and their malignant potential is difficult to assess even histologically [3,4,6,7,8]. Many cases prove benign but the general trend is towards volume increase and malignant transformation [4,5,6,7,8]. In a series of 36 patients with malignant PEComa, 20% had metastases, especially in the lungs or liver, but also in the nodes or in the peritoneum [2]. Moreover, these metastases sometimes develop very late [3]. In 2005, Folpe et al. proposed morphological and histologic criteria to classify gynecological PEComas as benign, intermediate or malignant (Table 1) [2,3,7]. Nevertheless, the scarcity of published cases makes these conclusions risky and it is clear that this class of tumors requires much larger studies [3]. The recent increase in cases, linked to the increasing use of cross-sectional imaging and improved histological tumor recognition, certainly contribute in that direction.

**Table 1:** Classification of gynecological PEComas.

Category Criteria	

Benign	None of: Size ≥ 5 cm Infiltrative growth pattern High nuclear grade cellularity Mitotic rate > 1/50 high power fields Necrosis Vascular invasion
Uncertain malignant potential	One of: Nuclear pleomorphism Multinucleated giant cell Size ≥ 5 cm
Malignant	Two or more: Size ≥ 5cm Infiltrative growth pattern High nuclear grade cellularity Mitotic rate > 1/50 high power fields Necrosis Vascular invasion

The symptomatology of PEComas is non-specific, the more as the tumor topography proves variable. Regarding uterine tumors, if the smallest lesions are often discovered incidentally, larger masses may trigger symptoms that range from vaginal bleeding to hemoperitoneum via abdominal discomfort, whose etiology is so vague [1,3].

PEComas are detected by cross-sectional imaging but only histologic and immunohistochemical analysis enables accurate diagnosis [2,4,9]. Ultrasound usually detects an inhomogeneous mass whereas CT scans reveal a rather well-defined hypo- or isointense structure, capturing iodinated contrast media. Necrosis or hemorrhage areas and calcifications can be identified, especially in malignant cases [2]. MRI also displays a rather well-circumscribed mass, in hypo- or isointenseT1 signal and in hyperintense T2 signal relative to healthy muscles, showing a high uptake of gadolinium. Fluoro-deoxyglucose PET scanner seems to be mainly useful in the detection of possible metastases and for the follow-up of treated patients [10]. Whatever the type of cross-sectional imaging used, PEComas remain indistinguishable from other uterine tumors such as leiomyomas, sarcomas or carcinomas, to mention but a few, and the following case is a clear illustration of this [9].

A 25-year-old nulligravid woman consults her gynecologist for metrorrhagia. Clinical examination suspects a pelvic mass and endovaginal ultrasonography reveals a granulomatous ovoid uterine tumor of 10 cm in the long axis, with a rich vascular network at color Doppler and Doppler arterial spectra of low resistance-type (Fig. 1). The right and left ovaries have shifted laterally and cranially respectively but remain normal looking. Magnetic examination confirms a clearly defined uterine wall tumor, of comparable size, in hypointense T1 signal, in hyperintense T2 signal mimicking a “snowstorm” and avidly capturing gadolinium with a light washout on the delayed phase (Figs. 2,3). A hydatidiform mole is suspected but the intramural topography of the tumor and the normality of the HCG test do not really argue in favor of this hypothesis. The patient benefits from inter-ovarian hysterectomy with cryopreservation of ovarian tissue for preservation of her subsequent fertility. The histologic analysis reports an intermediate malignant PEComa. At present, the young woman is alive and well and will be followed up on a regular basis.

**Figure 1:**
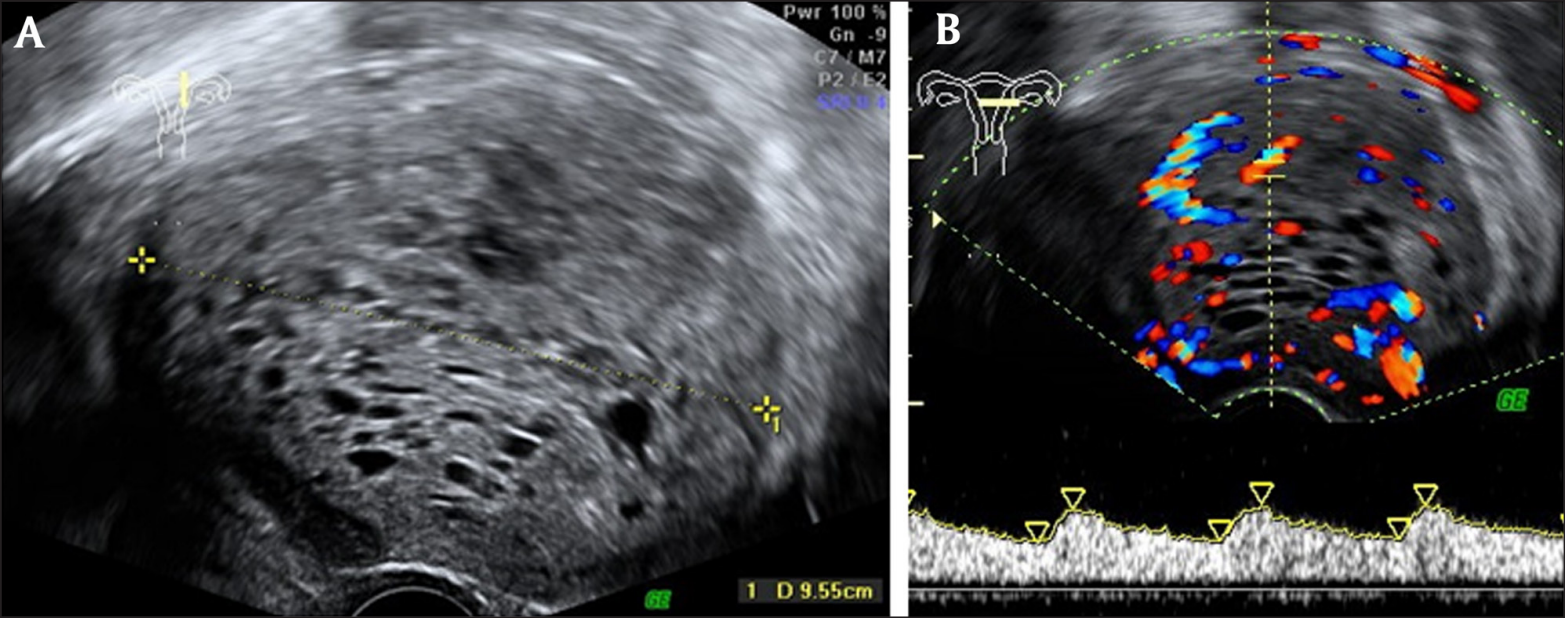
**(A)** Endovaginal ultrasound: the nearly 10 cm multicystic mass between the yellow crosses. **(B)** Color Doppler endovaginal ultrasound: the tumor appears hypervascular with low resistance arterial spectra (yellow arrows).

**Figure 2:**
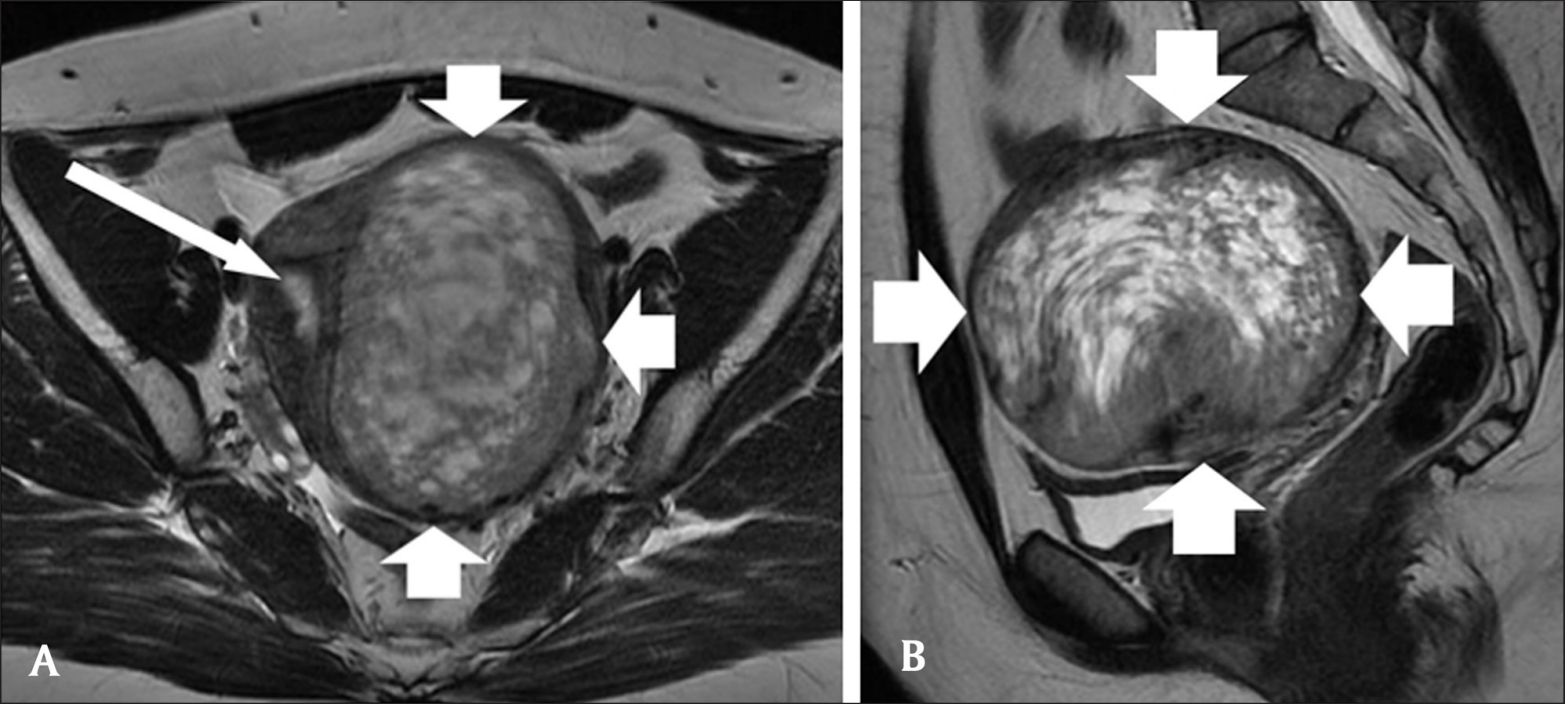
**(A)** Axial T2-weighted MR image: the hyperintense «snowstorm» ovoïd mass (thick arrows) and the uterine lumen (thin arrow). **(B)** Sagittal T2-weighted MR image: the tumor (arrows).

**Figure 3:**
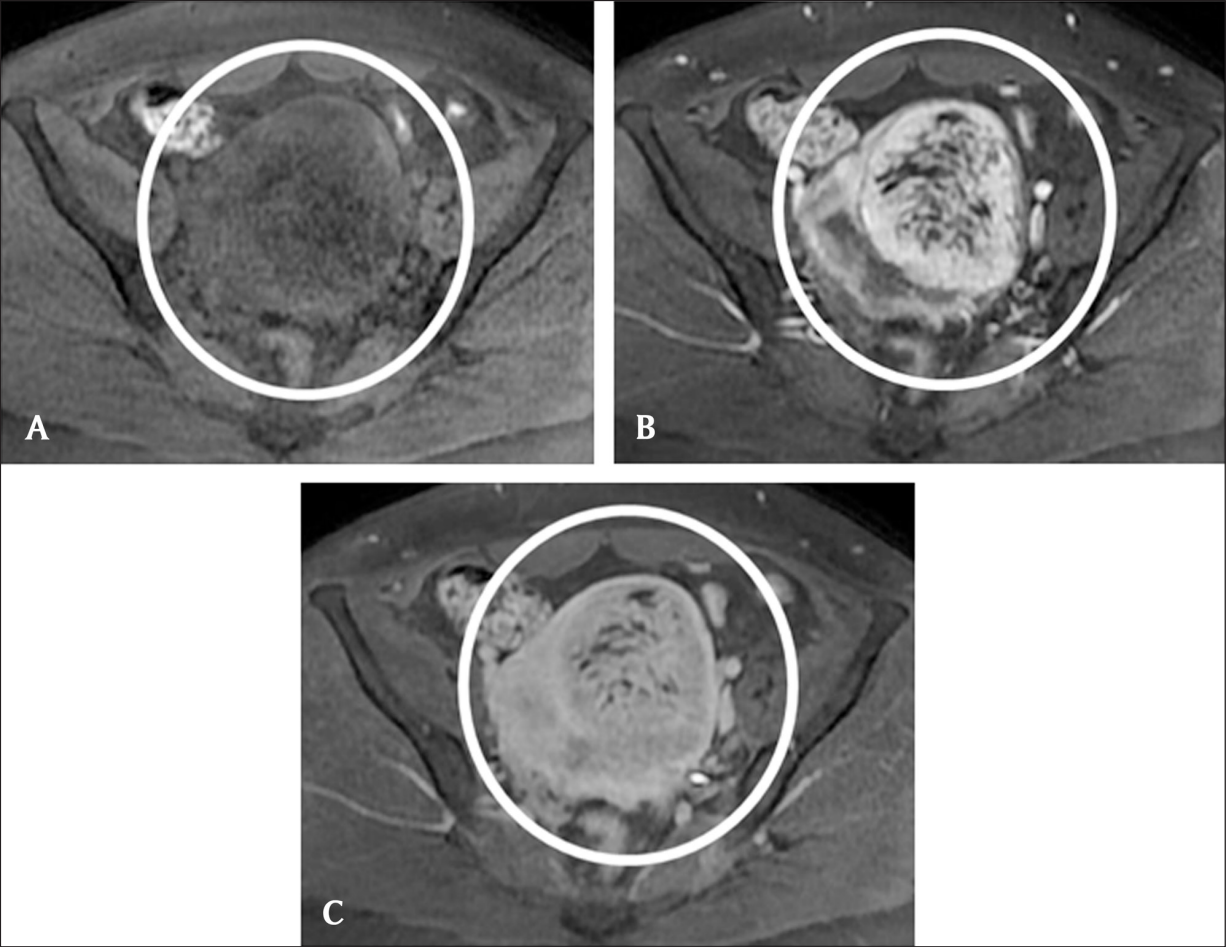
**(A)** Unenhanced axial T1-weighted MR image: the hypointense uterine lesion in the center of The circle. **(B)** Enhanced axial T1-weighted MR image: the arterial phase shows avid captation. **(C)** Enhanced axial T1-weighted MR image: the light tumoral washout on the delayed phase.

The therapeutic strategy with respect to PEComas is poorly established because of the rarity of their occurrence [5,6,7,8]. For small tumors (less than 5 cm in diameter), according to the criteria of Folpe, observation over the long term can be proposed [4]. As for larger ones, the current trend is surgical resection when possible [3,7]. Adjuvant chemotherapy as well as radiotherapy proved disappointing in malignant cases [3,7]. The increased frequency of PEComas among female patients has suggested, in particular in case of metastases, the use of anti-hormone therapy and among others letrozole, which decreases the level of circulating estrogens [6]. Finally, sirolimus, or rapamycin, the inhibitor of mTOR, an enzyme which regulates cell proliferation, appears promising in the case of a generalization of the PEComa [3,6,7].

When a tumor is detected, the radiologist must keep in mind the possibility of a PEComa, even if the entity is rare. The definitive diagnosis of the tumor is based on the histologic and immunohistochemical study. The ability of this latter examination to define the benign or malignant character of the lesion remains, however, far from being absolute. The treatment of PEComas extends from active surveillance to surgery; anti-hormone treatments or the use of sirolimus should be offered in case of metastases. Larger and randomized studies are clearly needed to improve both the accurate determination of the malignant potential of PEComa and the therapeutic strategy to be applied.

## Competing Interests

The authors declare that they have no competing interests.
